# Bis(2-ethyl-1*H*-imidazole-κ*N* ^3^)bis­(nitrito-κ^2^ *O*,*O*′)copper(II) dihydrate

**DOI:** 10.1107/S1600536811022367

**Published:** 2011-06-18

**Authors:** Run-Qiang Zhu

**Affiliations:** a Ordered Matter Science Research Center, College of Chemistry and Chemical Engineering, Southeast University, Nanjing 211189, People’s Republic of China

## Abstract

In the title compound, [Cu(NO_2_)_2_(C_5_H_8_N_2_)]·2H_2_O, the Cu^2+^ ion exhibits site symmetry 2 and is hexacoordinated by four O atoms from two nitrite ions and two N atoms from two 2-ethyl-1*H*-imidazole mol­ecules. A free water mol­ecule assists in forming a three-dimensional network holding together the complexes *via* O—H⋯N, O—H⋯O and N—H⋯O hydrogen bonds.

## Related literature

For general background on ferroelectric compounds with metal–organic framework structures, see: Fu *et al.* (2009 ▶); Ye *et al.* (2006 ▶); Zhang *et al.* (2008 ▶, 2010 ▶). For graph-set motifs of hydrogen bonds, see: Bernstein *et al.* (1995 ▶). 
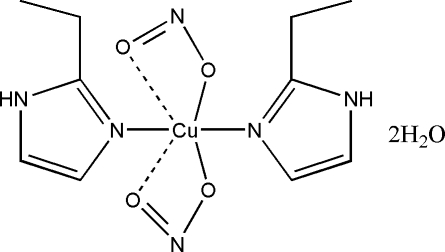


## Experimental

### 

#### Crystal data

[Cu(NO_2_)_2_(C_5_H_8_N_2_)]·2H_2_O
*M* *_r_* = 383.86
Orthorhombic, 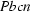

*a* = 12.960 (6) Å
*b* = 17.635 (7) Å
*c* = 7.288 (3) Å
*V* = 1665.7 (12) Å^3^
*Z* = 4
Mo *K*α radiation
μ = 1.35 mm^−1^
*T* = 293 K
0.30 × 0.25 × 0.20 mm

#### Data collection

Rigaku SCXmini diffractometer
Absorption correction: multi-scan (*CrystalClear*; Rigaku, 2005 ▶) *T*_min_ = 0.674, *T*_max_ = 0.763
16649 measured reflections
1902 independent reflections
1712 reflections with *I* > 2σ(*I*)
*R*_int_ = 0.032

#### Refinement

*R*[*F* ^2^ > 2σ(*F* ^2^)] = 0.031
*wR*(*F* ^2^) = 0.079
*S* = 1.10
1902 reflections
113 parameters
5 restraints
H atoms treated by a mixture of independent and constrained refinement
Δρ_max_ = 0.33 e Å^−3^
Δρ_min_ = −0.24 e Å^−3^

### 

Data collection: *CrystalClear* (Rigaku, 2005 ▶); cell refinement: *CrystalClear*; data reduction: *CrystalClear*; program(s) used to solve structure: *SHELXS97* (Sheldrick, 2008 ▶); program(s) used to refine structure: *SHELXL97* (Sheldrick, 2008 ▶); molecular graphics: *SHELXTL* (Sheldrick, 2008 ▶); software used to prepare material for publication: *SHELXL97*.

## Supplementary Material

Crystal structure: contains datablock(s) I, global. DOI: 10.1107/S1600536811022367/vn2010sup1.cif

Structure factors: contains datablock(s) I. DOI: 10.1107/S1600536811022367/vn2010Isup2.hkl

Additional supplementary materials:  crystallographic information; 3D view; checkCIF report

## Figures and Tables

**Table 1:** Selected bond lengths (Å)

Cu1—N1	1.9752 (17)
Cu1—O6	2.0255 (15)
Cu1—O5	2.4501 (18)

**Table 2:** Hydrogen-bond geometry (Å, °)

*D*—H⋯*A*	*D*—H	H⋯*A*	*D*⋯*A*	*D*—H⋯*A*
N2—H2*B*⋯O7	0.86	1.99	2.830 (3)	167
O7—H1⋯O6^ii^	0.84 (1)	2.05 (2)	2.862 (2)	163 (3)
O7—H2⋯N3^iii^	0.83 (1)	2.18 (1)	3.004 (3)	167 (3)

Symmetry codes: (ii) 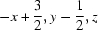; (iii) 
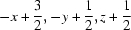
.

**Fig. 1.**
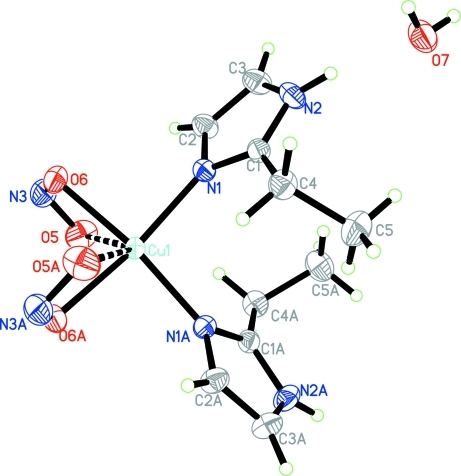
The molecular structure of the title compound, with the displacement ellipsoids drawn at the 30% probability level. The weak Cu—O interactions and the hydrogen bonds are shown as dashed lines. [Symmetry code: (A) - 1-x, y, 1/2-z]

**Fig. 2.**
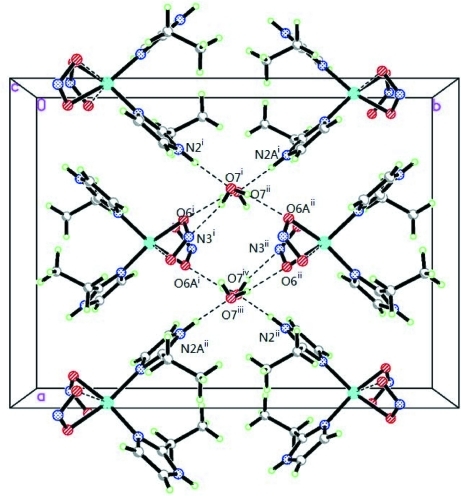
A view of the N—H···O, O—H···N and O—H···O interactions (dotted lines) in the crystal structure of the title compound. [Symmetry codes: (i)-x + 3/2, y - 1/2, z (ii)-x + 3/2, -y + 1/2, z + 1/2]

## supplementary crystallographic information

### Comment

We synthesized the title compound with the aim to find new ferroelectric materials (Fu *et al.*, 2009; Ye *et al.*, 2006; Zhang *et al.*, 2008; Zhang *et al.*, 2010). For the title compound no dielectric anomalies were observed in the range from 190 K to near its melting point (m.p. >400 K). A view of the title compound is shown in Fig. 1. The structure is consolidated by multiple intermolecular and intramolecular hydrogen bonds between O and N. This hydrogen bonding (Table 1, Fig. 2) produces a three-dimensional network. Hydrogen bonding is the most reliable design element in the non-covalent assembly of neutral molecules with donor and acceptor functionalities, and as such it is the most important interaction in crystal engineering (Bernstein *et al.*, 1995). The two contact distances between Cu and the oxygens of the nitrate ion  are very different (Cu1–O5=2.4501 (18) Å and Cu1–O6=2.0255 (15) Å), showing thus only moderate bonding.

### Experimental

An aqueous solution of 2-ethyl imidazole (2.4 g, 25 mmol) and H_2_SO_4_(12.5 mmol) was treated with CuSO_4_ (250 g, 12.5 mmol). After the mixture was churned for a few minutes, Ba(NO_2_)_2_ (6.18 g, 25 mmol) was added to give a blue solution. Slow evaporation of the solution yielded blue crystals after a few days.

### Refinement

Positional parameters of all H atoms except H1 and H2 were calculated geometrically and the H atoms were set to ride the C atoms and N atoms to which they are bonded, with *U*_iso_(H)= 1.2 *U*_iso_(C, N) and 1.5 *U*_iso_(C) for methyl H atoms. The H atoms of the water molecule  were restrained with O—H = 0.84 Å yielding O7—H1 = 0.835 (11) Å  and O7—H2 = 0.834 (11) Å.

### Crystal data

**Table:**

[Cu(NO_2_)_2_(C_5_H_8_N_2_)]·2H_2_O	*F*(000) = 796
*M**_r_* = 383.86	*D*_x_ = 1.531 Mg m^−^^3^
Orthorhombic, *P**b**c**n*	Mo *K*α radiation, λ = 0.71073 Å
Hall symbol: -P 2n 2ab	Cell parameters from 3875 reflections
*a* = 12.960 (6) Å	θ = 2.8–27.5°
*b* = 17.635 (7) Å	µ = 1.35 mm^−^^1^
*c* = 7.288 (3) Å	*T* = 293 K
*V* = 1665.7 (12) Å^3^	Block, blue
*Z* = 4	0.30 × 0.25 × 0.20 mm

### Data collection

**Table:**

Rigaku, SCXmini diffractometer	1902 independent reflections
Radiation source: fine-focus sealed tube	1712 reflections with *I* > 2σ(*I*)
graphite	*R*_int_ = 0.032
ω scans	θ_max_ = 27.5°, θ_min_ = 3.6°
Absorption correction: multi-scan (*CrystalClear*; Rigaku, 2005)	*h* = −16→16
*T*_min_ = 0.674, *T*_max_ = 0.763	*k* = −22→22
16649 measured reflections	*l* = −9→9

### Refinement

**Table:**

Refinement on *F*^2^	Primary atom site location: structure-invariant direct methods
Least-squares matrix: full	Secondary atom site location: difference Fourier map
*R*[*F*^2^ > 2σ(*F*^2^)] = 0.031	Hydrogen site location: inferred from neighbouring sites
*wR*(*F*^2^) = 0.079	H atoms treated by a mixture of independent and constrained refinement
*S* = 1.10	*w* = 1/[σ^2^(*F*_o_^2^) + (0.0424*P*)^2^ + 0.3653*P*] where *P* = (*F*_o_^2^ + 2*F*_c_^2^)/3
1902 reflections	(Δ/σ)_max_ = 0.001
113 parameters	Δρ_max_ = 0.33 e Å^−^^3^
5 restraints	Δρ_min_ = −0.24 e Å^−^^3^

### Special details

**Table:**

Geometry. All e.s.d.'s are estimated using the full covariance matrix. The cell e.s.d.'s are taken into account individually in the estimation of e.s.d.'s in distances and angles; correlations between e.s.d.'s in cell parameters are only used when they are defined by crystal symmetry.
Refinement. Refinement of *F*^2^ against ALL reflections. The weighted *R*-factor *wR* and goodness of fit *S* are based on *F*^2^, conventional *R*-factors *R* are based on *F*, with *F* set to zero for negative *F*^2^. The threshold expression of *F*^2^ > σ(*F*^2^) is used only for calculating *R*-factors(gt) *etc*. and is not relevant to the choice of reflections for refinement. *R*-factors based on *F*^2^ are statistically about twice as large as those based on *F*, and *R*- factors based on ALL data will be even larger.

### Fractional atomic coordinates and isotropic or equivalent isotropic displacement parameters (Å^2^)

**Table:**

	*x*	*y*	*z*	*U*_iso_*/*U*_eq_	
C1	0.65105 (15)	0.16593 (12)	0.2425 (3)	0.0354 (4)	
C2	0.63010 (17)	0.21323 (12)	−0.0306 (3)	0.0429 (5)	
H2A	0.6067	0.2436	−0.1262	0.051*	
C3	0.69903 (18)	0.15687 (14)	−0.0455 (3)	0.0502 (6)	
H3A	0.7321	0.1411	−0.1521	0.060*	
C4	0.64560 (18)	0.15017 (13)	0.4424 (3)	0.0451 (5)	
H4A	0.6091	0.1914	0.5021	0.054*	
H4B	0.7151	0.1488	0.4917	0.054*	
C5	0.5918 (2)	0.07583 (15)	0.4881 (4)	0.0637 (7)	
H5A	0.5923	0.0683	0.6185	0.096*	
H5B	0.6274	0.0347	0.4293	0.096*	
H5C	0.5218	0.0776	0.4453	0.096*	
N1	0.59959 (13)	0.21864 (9)	0.1508 (2)	0.0342 (4)	
N2	0.71115 (13)	0.12722 (11)	0.1257 (3)	0.0465 (4)	
H2B	0.7508	0.0899	0.1541	0.056*	
Cu1	0.5000	0.292843 (17)	0.2500	0.02994 (13)	
O6	0.57539 (11)	0.37719 (8)	0.1173 (2)	0.0460 (4)	
O7	0.82902 (14)	0.00761 (11)	0.2776 (3)	0.0563 (5)	
O5	0.44637 (13)	0.34927 (11)	−0.0420 (2)	0.0604 (5)	
N3	0.52032 (17)	0.39184 (12)	−0.0252 (3)	0.0528 (5)	
H1	0.848 (2)	−0.0289 (14)	0.213 (4)	0.088 (12)*	
H2	0.8774 (18)	0.0299 (17)	0.330 (5)	0.104 (13)*	

### Atomic displacement parameters (Å^2^)

**Table:**

	*U*^11^	*U*^22^	*U*^33^	*U*^12^	*U*^13^	*U*^23^
C1	0.0307 (9)	0.0311 (10)	0.0443 (11)	−0.0003 (8)	−0.0020 (8)	−0.0012 (8)
C2	0.0446 (12)	0.0477 (12)	0.0364 (11)	0.0071 (9)	0.0052 (9)	0.0002 (9)
C3	0.0488 (12)	0.0564 (14)	0.0455 (13)	0.0086 (10)	0.0094 (10)	−0.0069 (11)
C4	0.0462 (11)	0.0459 (12)	0.0434 (12)	0.0009 (9)	−0.0055 (10)	0.0050 (10)
C5	0.0735 (17)	0.0546 (15)	0.0631 (16)	−0.0072 (13)	0.0031 (14)	0.0163 (12)
N1	0.0349 (8)	0.0324 (8)	0.0352 (9)	0.0022 (6)	0.0032 (7)	0.0002 (7)
N2	0.0405 (9)	0.0434 (10)	0.0557 (12)	0.0136 (8)	0.0012 (9)	−0.0035 (9)
Cu1	0.0318 (2)	0.0253 (2)	0.0327 (2)	0.000	0.00267 (12)	0.000
O6	0.0511 (8)	0.0358 (7)	0.0512 (9)	−0.0066 (6)	0.0063 (7)	0.0036 (7)
O7	0.0532 (10)	0.0438 (10)	0.0720 (12)	0.0097 (8)	−0.0056 (9)	−0.0079 (9)
O5	0.0549 (10)	0.0723 (12)	0.0541 (10)	−0.0012 (9)	−0.0072 (8)	0.0181 (9)
N3	0.0633 (13)	0.0455 (11)	0.0497 (12)	0.0011 (9)	0.0107 (10)	0.0131 (9)

### Geometric parameters (Å, °)

**Table:**

C1—N1	1.325 (3)	C5—H5B	0.9600
C1—N2	1.341 (3)	C5—H5C	0.9600
C1—C4	1.485 (3)	N1—Cu1	1.9752 (17)
C2—C3	1.341 (3)	N2—H2B	0.8600
C2—N1	1.383 (3)	Cu1—N1^i^	1.9752 (17)
C2—H2A	0.9300	Cu1—O6	2.0255 (15)
C3—N2	1.362 (3)	Cu1—O6^i^	2.0255 (15)
C3—H3A	0.9300	Cu1—O5	2.4501 (18)
C4—C5	1.522 (3)	O6—N3	1.287 (3)
C4—H4A	0.9700	O7—H1	0.835 (11)
C4—H4B	0.9700	O7—H2	0.834 (11)
C5—H5A	0.9600	O5—N3	1.223 (3)
			
N1—C1—N2	109.23 (18)	C1—N1—C2	106.86 (17)
N1—C1—C4	127.04 (19)	C1—N1—Cu1	127.51 (14)
N2—C1—C4	123.73 (19)	C2—N1—Cu1	125.63 (14)
C3—C2—N1	108.6 (2)	C1—N2—C3	108.61 (18)
C3—C2—H2A	125.7	C1—N2—H2B	125.7
N1—C2—H2A	125.7	C3—N2—H2B	125.7
C2—C3—N2	106.7 (2)	N1^i^—Cu1—N1	97.01 (10)
C2—C3—H3A	126.7	N1^i^—Cu1—O6	167.58 (7)
N2—C3—H3A	126.7	N1—Cu1—O6	89.80 (7)
C1—C4—C5	113.4 (2)	N1^i^—Cu1—O6^i^	89.80 (7)
C1—C4—H4A	108.9	N1—Cu1—O6^i^	167.58 (7)
C5—C4—H4A	108.9	O6—Cu1—O6^i^	85.48 (9)
C1—C4—H4B	108.9	N1^i^—Cu1—O5	113.70 (7)
C5—C4—H4B	108.9	N1—Cu1—O5	97.84 (7)
H4A—C4—H4B	107.7	O6—Cu1—O5	54.81 (6)
C4—C5—H5A	109.5	O6^i^—Cu1—O5	88.83 (7)
C4—C5—H5B	109.5	N3—O6—Cu1	105.39 (13)
H5A—C5—H5B	109.5	H1—O7—H2	113.6 (19)
C4—C5—H5C	109.5	N3—O5—Cu1	86.57 (13)
H5A—C5—H5C	109.5	O5—N3—O6	113.08 (18)
H5B—C5—H5C	109.5		

Symmetry codes: (i) −*x*+1, *y*, −*z*+1/2.

### Hydrogen-bond geometry (Å, °)

**Table:**

*D*—H···*A*	*D*—H	H···*A*	*D*···*A*	*D*—H···*A*
N2—H2B···O7	0.86	1.99	2.830 (3)	167.
O7—H1···O6^ii^	0.84 (1)	2.05 (2)	2.862 (2)	163 (3)
O7—H2···N3^iii^	0.83 (1)	2.18 (1)	3.004 (3)	167 (3)

Symmetry codes: (ii) −*x*+3/2, *y*−1/2, *z*; (iii) −*x*+3/2, −*y*+1/2, *z*+1/2.
